# Quantitative Kinetic Study of the Actin-Bundling Protein L-Plastin and of Its Impact on Actin Turn-Over

**DOI:** 10.1371/journal.pone.0009210

**Published:** 2010-02-15

**Authors:** Ziad Al Tanoury, Elisabeth Schaffner-Reckinger, Aliaksandr Halavatyi, Céline Hoffmann, Michèle Moes, Ermin Hadzic, Marie Catillon, Mikalai Yatskou, Evelyne Friederich

**Affiliations:** 1 Laboratory of Cytoskeleton and Cell Plasticity, Life Sciences Research Unit, University of Luxembourg, Luxembourg City, Luxembourg; 2 Laboratory of Plant Molecular Biology, Public Research Center for Health (CRP-Santé), Strassen, Luxembourg; University of Birmingham, United Kingdom

## Abstract

**Background:**

Initially detected in leukocytes and cancer cells derived from solid tissues, L-plastin/fimbrin belongs to a large family of actin crosslinkers and is considered as a marker for many cancers. Phosphorylation of L-plastin on residue Ser5 increases its F-actin binding activity and is required for L-plastin-mediated cell invasion.

**Methodology/Principal Findings:**

To study the kinetics of L-plastin and the impact of L-plastin Ser5 phosphorylation on L-plastin dynamics and actin turn-over in live cells, simian Vero cells were transfected with GFP-coupled WT-L-plastin, Ser5 substitution variants (S5/A, S5/E) or actin and analyzed by fluorescence recovery after photobleaching (FRAP). FRAP data were explored by mathematical modeling to estimate steady-state reaction parameters. We demonstrate that in Vero cell focal adhesions L-plastin undergoes rapid cycles of association/dissociation following a two-binding-state model. Phosphorylation of L-plastin increased its association rates by two-fold, whereas dissociation rates were unaffected. Importantly, L-plastin affected actin turn-over by decreasing the actin dissociation rate by four-fold, increasing thereby the amount of F-actin in the focal adhesions, all these effects being promoted by Ser5 phosphorylation. In MCF-7 breast carcinoma cells, phorbol 12-myristate 13-acetate (PMA) treatment induced L-plastin translocation to *de novo* actin polymerization sites in ruffling membranes and spike-like structures and highly increased its Ser5 phosphorylation. Both inhibition studies and siRNA knock-down of PKC isozymes pointed to the involvement of the novel PKC-δ isozyme in the PMA-elicited signaling pathway leading to L-plastin Ser5 phosphorylation. Furthermore, the L-plastin contribution to actin dynamics regulation was substantiated by its association with a protein complex comprising cortactin, which is known to be involved in this process.

**Conclusions/Significance:**

Altogether these findings quantitatively demonstrate for the first time that L-plastin contributes to the fine-tuning of actin turn-over, an activity which is regulated by Ser5 phosphorylation promoting its high affinity binding to the cytoskeleton. In carcinoma cells, PKC-δ signaling pathways appear to link L-plastin phosphorylation to actin polymerization and invasion.

## Introduction

Cell motility is driven by remodeling of the actin cytoskeleton and cell contacts with the extracellular matrix (ECM) [1], a process which is under the control of a plethora of actin-binding proteins. In particular, actin filament crosslinkers have been proposed to play a critical role in the organization and dynamics of the actin cytoskeleton and its cellular functions.

L-plastin (also termed L-fimbrin), the hematopoietic plastin isoform, was initially detected in leukocytes [2]. Aberrantly expressed in cancer cells derived from solid tissues [3]–[7], L-plastin promotes invasion of cultured epithelial cells supporting its role in cancer progression [8], [9]. L-plastin is a representative member of a large family of actin-crosslinking or -bundling proteins, including α-actinin and filamin [10]. Members of this family share a conserved ∼250 amino acid F-actin binding domain (ABD) [11] which is composed of two tandemly arranged calponin-homology (CH) domains [12]. Plastins contain two ABDs which are packed into a compact fold [13], [14] enabling them to organize actin filaments into tight bundles [15], as well as an amino-terminal calmodulin-like headpiece that comprises two Ca^2+^-binding EF-hand modules [16]. In cells, L-plastin localizes to various actin-rich membrane structures involved in locomotion, adhesion, signaling and immune defense, including focal adhesions, podosomes, filopodia and the phagocytic cup, thus supporting a role for L-plastin in the organization of the actin cytoskeleton and in signal transduction [9], [17]–[19]. Biochemical *in vitro* data have shown that L-plastin not only organizes filaments into arrays but also prevents them from depolymerization suggesting that it may regulate their turn-over [20]. Further evidence for a role in the control of actin turn-over is provided by the observation that L-plastin could substitute for yeast plastin in a *Sac6* null mutant which exhibited defects in actin polymerization [21].

Among the three human plastin isoforms which also include T- and I-plastin, only L-plastin has been reported to be regulated through phosphorylation [22] in response to signals triggering the activation of the immune response, cell migration and proliferation. Phosphorylation on residue serine-5 (Ser5), the major L-plastin phosphorylation site [22]–[24], has been shown to increase its F-actin-binding and -bundling activities *in vitro* and to be required for efficient targeting of L-plastin to focal adhesion sites as well as for cancer cell invasion [8], [9]. However, the impact of L-plastin Ser5 phosphorylation on L-plastin binding-unbinding kinetics and on actin turn-over in live cells remains to be investigated.

Distinct protein kinases appear to be responsible for L-plastin phosphorylation depending on the cell type and environment. In hematopoietic cells and in various other non-transformed cell types, it is well-established that L-plastin can be phosphorylated on residue Ser5 by the cAMP-dependent Protein Kinase A (PKA) which has also been shown to directly phosphorylate L-plastin *in vitro* [9], [24]. However, in addition to PKA, other kinases such as PKC have been suggested to contribute to L-plastin phosphorylation in leukocytes, fibroblasts and neutrophils [22], [25].

Here we studied L-plastin/actin kinetics in live cells and L-plastin phosphorylation in response to signals triggering cytoskeletal rearrangements. Quantitative fluorescence recovery after photobleaching (FRAP) assays revealed that L-plastin associated with focal adhesions following a two-binding-state model and that L-plastin phosphorylation increased its association rates and, hence, its capacity to stably dock to specific cytoskeleton structures. Importantly, L-plastin induced an increase of the F-actin content at focal adhesion sites by decreasing the actin dissociation rate. These effects on actin turn-over were considerably enhanced by L-plastin phosphorylation on residue Ser5. Furthermore, treatment of MCF-7 breast cancer cells with the cell invasion promoting agent phorbol 12-myristate 13-acetate (PMA), triggered the translocation of endogenous L-plastin to ruffling membranes and spike-like structures as well as L-plastin Ser5 phosphorylation, through activation of PKC-δ signaling pathways.

## Results

### Binding Kinetics of L-Plastin Follow a Two-Binding-State Model and Are Modulated by Ser5 Phosphorylation in Live Vero Cells

**Figure 1:**
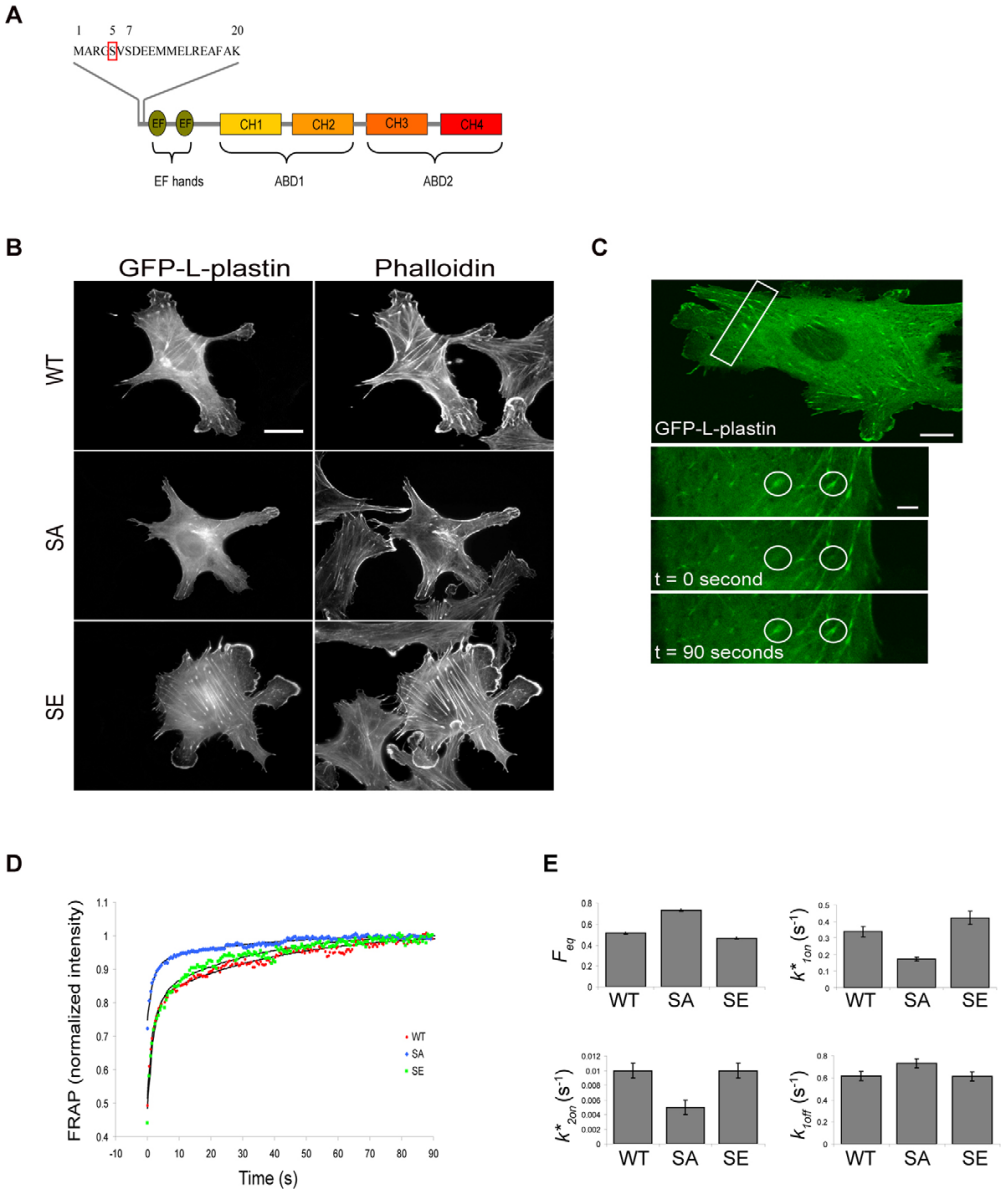
L-plastin phosphorylation modulates its mobility in focal adhesions. (A). Schematic representation of wild-type (WT) L-plastin showing the headpiece domain followed by two independent actin binding domains (ABDs). Residue serine-5 (Ser5) of the headpiece was mutated to alanine (SA) to generate unphosphorylatable L-plastin or to glutamic acid (SE) to generate an L-plastin variant mimicking constitutive phosphorylation. (B). Expression and localization of GFP-coupled L-plastin phosphorylation variants in Vero cells. Vero cells were transfected with cDNA encoding GFP-L-plastin phosphorylation variants. After 48 hours, cells were fixed and processed for immunofluorescence. The localization of L-plastin and F-actin was analyzed with an epifluorescence microscope (Leica DMRX microscope) after staining with Rhodamine-conjugated phalloidin to visualize polymerized actin. Scale bar, 20 µm. (C). A typical FRAP experiment carried out on a Vero cell transfected with WT GFP-L-plastin. The boxed region in the upper panel (scale bar, 10 µm) is shown enlarged in the bottom panels (scale bar, 4 µm). Circular spots, surrounded by a white line, are regions of interest (ROI) that are submitted to photobleaching and that have a diameter of 5 µm. Such spot size was selected to smooth local area effects and visually well-represents the focal adhesion region. Pictures were recorded before bleaching, immediately after bleaching and 90 seconds after bleaching. (D). Normalized FRAP recovery curves of wild type (WT, red), Ser5/Ala (SA, blue) and Ser5/Glu (SE, green) GFP-L-plastin fusions are compared to the curves predicted by the two-binding-state model (black curves). Data were obtained from three independent experiments representing 10 FRAP recordings for each condition. (E). Charts representing biochemical parameters obtained from fitting data with a two-binding-state model. Bars represent the mean ± s.d. *P*-values were calculated using standard Student's t-test. A *p-*value<0.05, considered as statistically significant, was obtained for *F_eq_*, k*_1on_ and k*_2on_ but not for *k_1off_*.

To investigate the steady-state dynamics of L-plastin and actin in living cells and the role of L-plastin phosphorylation on Ser5 herein (Fig. 1A), we performed confocal microscopy-based fluorescence recovery after photobleaching (FRAP) experiments using previously characterized L-plastin variants in fibroblast-like Vero cells which do not express endogenous L-plastin [9]. FRAP, which is a powerful approach for studying molecular mobility in live cells [26], [27] was combined with mathematical modeling to estimate the steady-state kinetics of L-plastin variants and actin turn-over which reflects actin polymerization and depolymerization reactions [28], [29]. Similar to epitope-tagged wild type L-plastin [9], wild type (WT-) L-plastin fused to GFP colocalized with actin in focal adhesions, membrane protrusions and along stress fibers, as visualised by epifluorescence microscopy (Fig. 1B, upper panels). To study the kinetics of the phosphorylated pool of L-plastin in Vero cells, we took advantage of the fact that transfected WT-L-plastin is phosphorylated on Ser5 and targeted to focal adhesions in these cells [9]. The bleach was therefore performed in a small region of interest (ROI) in focal adhesions (Fig. 1C). For each ROI, the experimental intensity recoveries were normalized and averaged. The obtained curves exhibited a fast and a slow phase of recovery (Fig. 1D). Twenty seconds after photobleaching, 89% of recovery was reached for WT GFP-L-plastin suggesting that the protein is highly mobile, undergoing rapid cycles of association and dissociation, as reported for other crosslinking proteins [26], [30]. Quantitatively, the best fits of the FRAP curves were obtained with a “two-binding-state” model [31]. This model may be applied to a system in which a molecule exhibits two distinct binding states involved in the interaction with the free binding sites of a partner molecule to form a complex. Analysis with the two-binding-state model allowed the separation of FRAP recovery curves into two largely independent phases, a first relatively quick phase from zero to ten seconds (*k_1off_* = 0.616 s^−1^ for WT) and a second much slower phase that represented the plateau (*k_2off_* = 0.03 s^−1^ for WT), such that *k_1off _*≫* k_2off_*. Based on this model, we estimated the equilibrium normalized concentration of free WT-L-plastin molecules *F_eq_* (*F_eq_*_(WT)_ = 0.513±0.01) and the association rates k*_1on_ (k*_1on__(WT)_ = 0.338±0.032 s^−1^) and k*_2on_ (k*_2on__(WT)_ = 0.01±0.001 s^−1^) by fitting the normalized experimental FRAP curves with equation (5) (Fig. 1E, see Materials and Methods).

To estimate how phosphorylation of L-plastin affects these parameters, we used two previously characterized phosphorylation variants of L-plastin in which residue Ser5 of L-plastin was replaced with an alanine (L-plastin Ser5/Ala) or a glutamate residue (L-plastin Ser5/Glu), to inactivate or to mimic phosphorylation respectively [9]. In agreement with previous results obtained with epitope-tagged variants, a comparable yet more pronounced localization to F-actin structures was observed with Ser5/Glu (S5/E) GFP-L-plastin as compared to WT GFP-L-plastin. In contrast, Ser5/Ala (S5/A) GFP-L-plastin exhibited a diffuse cytoplasmic staining with merely a weak localization in focal adhesions and membrane protrusions (Fig. 1B).

The recovery curve of GFP-coupled S5/A-L-plastin varied from that of WT- and S5/E-L-plastin. Indeed the S5/A variant exhibited a faster recovery curve as compared to WT and S5/E variants suggesting a higher mobility of this variant (96% of recovery twenty seconds after photobleaching) (Fig. 1D). This difference was unlikely to be merely due to fast molecule diffusion, as an additional set of FRAP experiments, in which the size of the bleached region was increased at least three-fold, yielded similar recoveries (data not shown). Calculation of *F_eq_* confirmed that, for the GFP-coupled S5/A-L-plastin variant, more unbound molecules were observed at the equilibrium than for the WT or S5/E GFP-L-plastin variants (Fig. 1E). The rate of association at the first ‘quick’ binding state k*_1on_ was two-fold lower for S5/A- as compared to WT-L-plastin (k*_1on__(WT)_ = 0.338±0.032 s^−1^; k*_1on__(SA)_ = 0.173±0.012 s^−1^), reflecting a lower association rate of the phosphorylation-defective S5/A variant. Conversely, the phosphomimetic S5/E-L-plastin variant exhibited a k*_1on_ which was even higher than that of the WT (k*_1on__(SE)_ = 0.42±0.04 s^−1^). Accordingly, the association rate at the second so-called ‘slow’ binding state k*_2on_ was also two-fold lower for S5/A-L-plastin as compared with WT- and S5/E-L-plastin variants which exhibited similar k*_2on_ association rates. Interestingly, WT-L-plastin and Ser5 substitution variants exhibited very similar dissociation rates at the quick-binding state *k_1off_* (*k_1off_*_(WT)_ = 0.616±0.041 s^−1^; *k_1off_*_(SA)_ = 0.073±0.041 s^−1^; *k_1off_*_(SE)_ = 0.612±0.042 s^−1^; see Fig. 1E) and at the slow-binding state *k_2off_* (data not shown).

Altogether, these findings suggest that the interaction of L-plastin with specific F-actin structures follows a “two-binding-state” model and that the association rates are up-regulated by L-plastin phosphorylation on residue Ser5.

### L-Plastin Modulates Actin Dynamics in a Phosphorylation-Dependent Manner in Vero Cells

**Figure 2:**
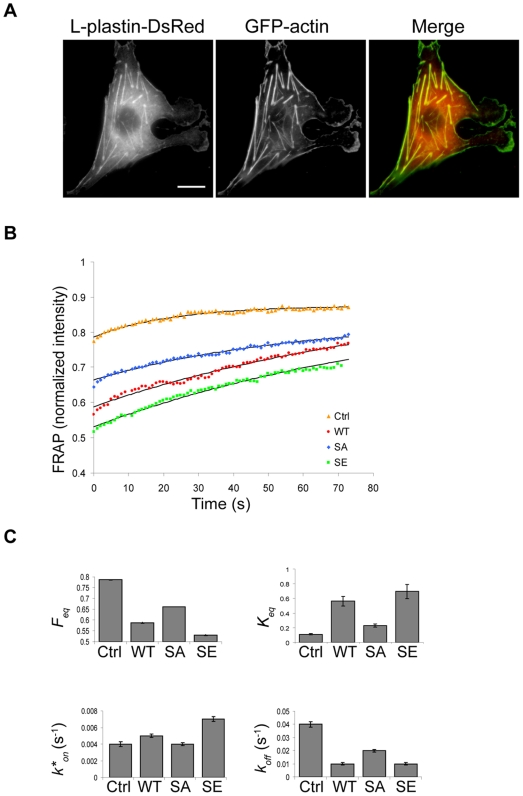
L-plastin phosphorylation modulates actin dynamics in focal adhesions. (A). Expression of L-plastin and actin in live Vero cells. Vero cells were cotransfected with monomeric L-plastin-DsRed-N1 fusion variants and GFP-actin. Wild type L-plastin-DsRed is shown here. Scale bar, 20 µm. (B). Normalized FRAP recovery curves obtained for actin in presence of wild type (WT, red), Ser5/Ala (SA, blue) and Ser5/Glu (SE, green) L-plastin-DsRed fusions or DsRed alone (control, orange) are compared to the curves predicted by the one-binding-state model (black curves). Data were obtained from three independent experiments representing 10 FRAP recordings for each condition. (C). Charts representing biochemical parameters obtained from data fitted with a one-binding-state model. Bars represent the mean ± s.d.

L-plastin appears to affect actin dynamics by binding along actin filaments, as supported by previous biochemical data [9], [20]. To address this issue, FRAP experiments were performed on GFP-actin in Vero cell focal adhesions, co-transfected with GFP-actin and L-plastin variants fused to monomeric DsRed (Fig. 2A). In agreement with previous kinetic studies of GFP-actin in cells [32], the fast recovery phase of GFP-actin involved rapid diffusion of the large free pool of actin monomers into the bleached area. Indeed, diffusion of the free monomers led to recovery in less than one second, which was below the characteristic time of the association/dissociation kinetics and time resolution of our experiments. The measured postbleach fluorescence intensity of GFP-actin was strongly reduced in WT-L-plastin expressing cells as compared to control cells (Fig. 2B), suggesting a decrease of the fraction of free diffusible actin monomers in response to L-plastin expression.

We applied a one-binding-state model in a reaction dominant regime to analyze our FRAP data [31]. This equation is applicable to various FRAP behaviours for a single binding reaction in the presence of fast monomer diffusion at steady-state. We estimated the equilibrium concentration of free actin monomers *F_eq_*, the dissociation rate *k_off_* and the association rate k*_on_ by fitting the normalized experimental FRAP curves with equation (3) (see Materials and Methods). We also calculated the ratio of bound to free G-actin molecules *K_eq_*, defined by 
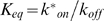
 [31]. These parameters provide information on how L-plastin variants influence the fraction of polymerized actin and the turn-over of actin filaments in focal adhesions. The estimated values for *F_eq_* revealed that the concentration of free actin monomers was clearly decreased in cells expressing WT-, S5/A- or S5/E-L-plastin-DsRed fusions when compared with control cells (Ctrl) expressing DsRed alone (*F_eq_*_(Ctrl)_ = 0.787±0.002; *F_eq_*_(WT)_ = 0.588±0.002; *F_eq_*_(SA)_ = 0.662±0.001; *F_eq_*_(SE)_ = 0.531±0.002) (Fig. 2C). Similarly, more F-actin was present at focal adhesion sites as illustrated by the *K_eq_* values (*K_eq_*_(Ctrl)_ = 0.112±0.009; *K_eq_*_(WT)_ = 0.563±0.064; *K_eq_*_(SA)_ = 0.23±0.021; *K_eq_*_(SE)_ = 0.694±0.098). This effect was more pronounced for the S5/E-L-plastin and less strong for the S5/A-L-plastin variants than for WT-L-plastin. Moreover the actin dissociation rate *k_off_* was affected in a comparable way by the three L-plastin variants, with a *k_off_* decrease of two-fold observed with the S5/A-L-plastin variant and a four-fold *k_off_* decrease obtained with WT- or S5/E-L-plastin (Fig. 2C). No significant changes in k*_on_ could be detected between S5/A-L-plastin- or WT-L-plastin-expressing cells and control cells, whereas the expression of the S5/E-L-plastin variant in cells led to a notable increase of the actin association rate k*_on_. Based on previous results, it can be excluded that the observed differences described above are due to dissimilar expression levels or stability of the phosphorylation variants [9].

Altogether, these data demonstrate that L-plastin affects actin dynamics and turn-over in focal adhesions by lowering the dissociation rate of actin, an effect which appears to be considerably enhanced by L-plastin Ser5 phosphorylation.

### PMA Induces Translocation of L-Plastin to *De Novo* Assembled Actin Structures and Enhances Its Ser5 Phosphorylation in MCF-7 Cells

**Figure 3:**
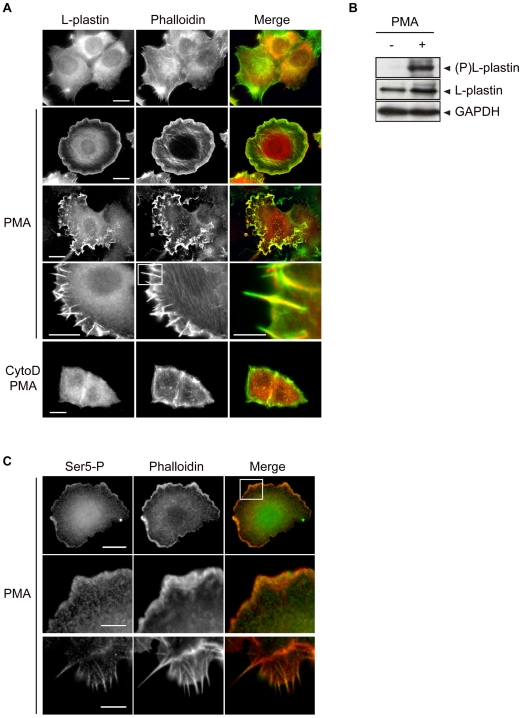
PMA induces the translocation of L-plastin to *de novo* assembled actin structures and triggers L-plastin phosphorylation. (A). PMA induces actin reorganization with concurrent local accumulation of L-plastin in actin-rich structures. MCF-7 cells were pretreated with or without 0.5 µM cytochalasin D (CytoD) and then treated with 1 µM PMA as indicated. The localization of L-plastin and F-actin was analyzed by epifluorescence microscopy after staining with an anti-L-plastin antibody and Alexa 488-conjugated phalloidin. The merged image of the boxed region indicated in the fourth row middle panel is shown enlarged on the right; scale bar, 2.5 µm. Other scale bars, 10 µm. (B). PMA induces phosphorylation of L-plastin on residue Ser5. MCF-7 cells were treated for 1 hour with or without 1 µM PMA at 37°C. Total cell extracts (50 µg) were analyzed by immunoblotting using antibodies specific for Ser5 phosphorylated L-plastin (anti-Ser5-P, upper panel), L-plastin (middle panel) or GAPDH (lower panel) to monitor equal protein loading. (C). Intracellular localization of Ser5 phosphorylated L-plastin in PMA-treated MCF-7 cells. MCF-7 cells treated for 1 h with 1 µM PMA were analyzed by epifluorescence microscopy after staining with an anti-Ser5-P antibody and Rhodamine-conjugated phalloidin. Upper panels illustrate the colocalization of Ser5 phosphorylated L-plastin with F-actin in ruffling membranes. Middle panels represent an enlarged detail of the cell shown in the upper panels (squared area). Lower panels represent a magnified detail of another cell showing the colocalization of Ser5 phosphorylated L-plastin with F-actin in spike-like structures. Scale bars, 10 µm (upper panels) and 2.5 µm (middle and lower panels).

Our results point to an important role for L-plastin in regulating actin dynamics, an activity which is promoted by Ser5 phosphorylation. In addition, it has been shown previously that Ser5 phosphorylation is required for L-plastin-mediated cell invasion [8], [9]. L-plastin phosphorylation in macrophages and leukocytes has been shown to be increased upon treatment with the phorbol ester PMA [24], [33], which is an invasion inducing agent that is generally used as a potent activator of classical (α, β, and γ) and novel (δ, ε, and η) PKC family members [34]–[36]. Here, we wanted to investigate the effects of PMA treatment in a cancer cell model, the MCF-7 breast carcinoma cell line. MCF-7 cells have been shown to express endogenous L-plastin although the expression level has been described as being heterogeneous [37]. Indeed, a fraction of MCF-7 cells highly expresses L-plastin, whereas other cells express L-plastin at a level close to or below immunofluorescence detection limits. Without PMA treatment, MCF-7 cells exhibited few prominent actin structures, as visualized by phalloidin staining, and L-plastin displayed a diffuse cytoplasmic distribution with a very weak localization to the cortical cytoskeleton (Fig. 3A, upper panels). PMA treatment of MCF-7 cells led to an important modification of the actin cytoskeleton organization and, in consequence, of the cell morphology. Interestingly, the phenotype of MCF-7 cells following PMA treatment was heterogeneous, with some cells exhibiting more or less pronounced ruffling membranes (Fig. 3A, second and third row panels) and others displaying protruding spike-like structures (Fig. 3A, fourth row panels). Surprisingly, L-plastin translocated to these newly assembled actin-rich structures in response to PMA treatment. Notably, L-plastin was mainly targeted to the proximal, membrane-embedded part of the protruding spike-like structures, whereas actin could be visualized throughout the entire length of the spikes. To discriminate between *de novo* actin polymerization and reorganization of existing actin filaments, cells were treated with the actin polymerization inhibitor cytochalasin D (CytoD), prior to the incubation with PMA. CytoD inhibited the PMA-induced actin reorganization and translocation of L-plastin, suggesting that PMA treatment induced *de novo* actin assembly rather than the reorganization of existing actin filaments (Fig. 3A, lower panels).

Next, a highly specific anti-serine-5 phosphorylated L-plastin (anti-Ser5-P) antibody, that had been characterized before [9], was used to determine L-plastin phosphorylation in immunoblotting experiments. Treatment of MCF-7 cells with PMA highly increased L-plastin phosphorylation (Fig. 3B), suggesting that PKC-elicited signaling upregulates L-plastin phosphorylation in these cells. Notably, the treatment of MCF-7 cells with a lower PMA concentration (0.1 µM) led to similar L-plastin translocation and phosphorylation events (data not shown).

Finally, we investigated the intracellular localization of Ser5 phosphorylated L-plastin in PMA-treated MCF-7 cells (Fig. 3C). Immunofluorescence analysis using the anti-Ser5-P antibody and Rhodamine-phalloidin revealed that L-plastin translocated to F-actin rich structures following PMA treatment was essentially the phosphorylated form. Indeed both ruffling membranes (upper and middle panels) and protruding spike-like structures formed at the cell periphery (lower panels) were stained with the anti-Ser5-P antibody. It is noteworthy that transfected S5/A-L-plastin also translocated to F-actin structures although to a lesser degree than WT- or S5/E-L-plastin, suggesting that phosphorylation is not strictly required for its translocation (data not shown). Altogether, these data support a PKC-dependent mechanism for L-plastin phosphorylation and its concomitant translocation to *de novo* assembled actin-rich structures in epithelial cancer cells, linking the regulation of L-plastin phosphorylation to that of actin dynamics.

### L-Plastin Associates with a Protein Complex Containing Cortactin, a Regulator of Actin Dynamics

**Figure 4:**
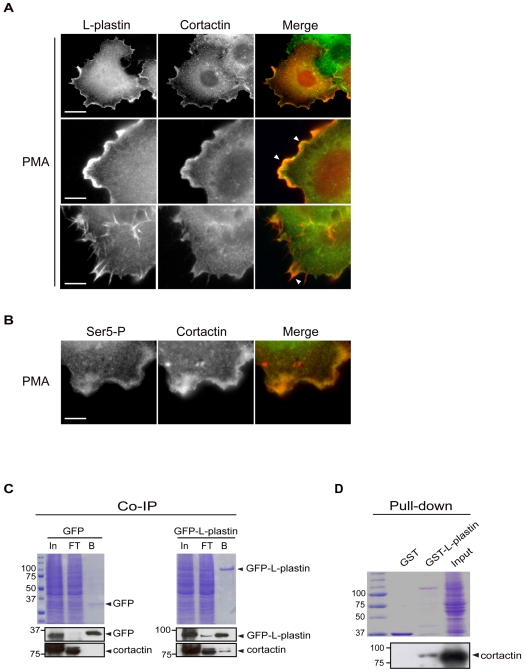
L-plastin associates with cortactin in protein complexes. (A). Colocalization of L-plastin with cortactin in MCF-7 cells. MCF-7 cells were treated with 1 µM PMA as described and analyzed by epifluorescence microscopy after staining with antibodies specific for L-plastin and cortactin. Immunofluorescence images illustrate the colocalization of L-plastin (red) and cortactin (green) in ruffling membranes (upper and middle panels) and at a lower extent in the membrane-embedded portion of spikes (lower panel). Arrows point to regions of colocalization. Scale bars, 10 µm (upper panels) and 3 µm (middle and lower panels). (B). Colocalization of serine-5 phosphorylated L-plastin with cortactin in MCF-7 cells. PMA-treated MCF-7 cells were stained with anti-Ser5-P and anti-cortactin antibodies and analyzed by epifluorescence microscopy. Serine-5 phosphorylated L-plastin (green) and cortactin (red) are colocalized in ruffling membranes. Scale bar, 3 µm. (C). Coimmunoprecipitation of cortactin with GFP-L-plastin in MCF-7 cells. GFP- or GFP-L-plastin-expressing MCF-7 cells were treated with PMA as described. Following cell lysis, protein extracts were subjected to immunoprecipitation with GFP-nanotrap. Aliquots of input [In], flow-through [FT], and bound fraction [B] were separated by SDS-PAGE and visualized either by Coomassie Blue staining (upper panels) or by immunoblot analysis using antibodies specific for GFP (middle panels) or cortactin (bottom panels). (D). Pull-down assay with cell extracts. GST and GST-L-plastin (20 µg) immobilized on glutathione-sepharose beads were incubated with untreated MCF-7 cell extracts (200 µg). The resulting complex was precipitated by centrifugation, separated by SDS-PAGE and visualized by Coomassie Blue staining (upper panel) or by immunoblotting using a cortactin-specific antibody (lower panel).

To biochemically corroborate these observations, we investigated whether L-plastin associates with protein complexes involved in the regulation of actin polymerization. We decided to use cortactin as a marker for sites of active actin polymerization. Cortactin, a Src-kinase protein substrate, which localizes to dynamic actin assembly sites such as lamellipodia, endosomes, podosomes and invadopodia [38], translocates to ruffling membranes upon PMA treatment [39]. As shown in figure 4A, a fraction of L-plastin colocalized with cortactin in PMA-treated MCF-7 cells, mainly in ruffling membranes (Fig. 4A, upper and middle panels) and, at a lower extent, in the membranous area where the spike-like structures protrude but not along the spikes themselves (Fig. 4A lower panels). Immunofluorescence analysis of PMA-treated cells using the anti-Ser5-P antibody provided evidence for the colocalization of phosphorylated L-plastin with cortactin in ruffling membranes (Fig. 4B).

Furthermore, we performed coimmunoprecipitation experiments with PMA-treated MCF-7 cells, using GFP-nanotrap [40]. This assay makes use of bead-linked monovalent lama antibodies directed against GFP and allows fast and efficient purification of GFP fusion proteins and their associated complexes formed in the cell. Importantly, immunoblot analysis revealed that cortactin efficiently coprecipitated with WT GFP-L-plastin extracted from PMA-treated cells (Fig. 4C). It is noteworthy that phosphorylation-defective S5/A GFP-L-plastin as well as WT GFP-L-plastin in PMA non-treated cells were both able to coimmunoprecipitate cortactin in some experiments (data not shown). These observations suggest that Ser5 phosphorylation is not an absolute requirement for binding, without excluding that it may modulate/promote L-plastin association with cytoskeletal protein complexes, as suggested by the FRAP data. Accordingly, pull-down assays with MCF-7 cell extracts using recombinant unphosphorylated GST-L-plastin also revealed cortactin in a complex with L-plastin (Fig. 4D). Altogether, our results identify L-plastin as a component of a protein complex comprising cortactin and provide further evidence for its recruitment to sites of active actin polymerization in cells.

### Novel PKC Isozymes Are Required for PMA-Induced Actin Cytoskeleton Rearrangements and L-Plastin Ser5 Phosphorylation

**Figure 5:**
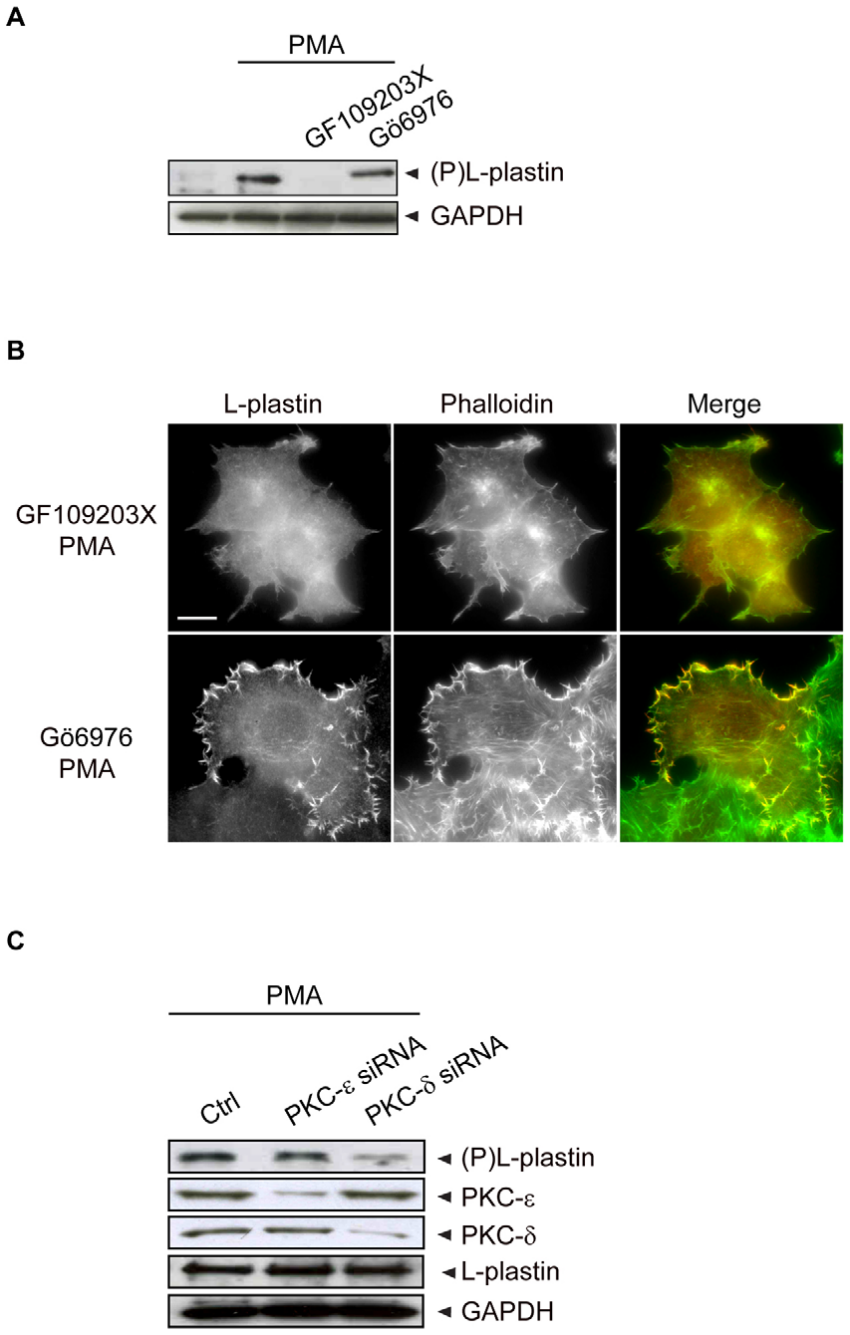
The novel PKC-δ isozyme is necessary for PMA-induced cytoskeleton reorganization and L-plastin Ser5 phosphorylation. (A). Novel PKC isozymes regulate PMA-induced L-plastin phosphorylation in MCF-7 cells. MCF-7 cells were pretreated for 3 hours with 5 µM of GF109203X (specific for α, β1, δ, ε, and ζ) or 0.5 µM of Gö6976 (specific for α and β1) and then treated for 1 hour with or without 1 µM PMA at 37°C. Total cell extracts (50 µg) were analyzed by immunoblotting using anti-Ser5-P L-plastin (upper panel) or anti-GAPDH (lower panel) antibodies. (B). PMA-induced actin cytoskeleton remodeling and L-plastin translocation involves novel PKC isozymes. MCF-7 cells were pretreated for 3 hours with 5 µM GF109203X or 0.5 µM Gö6976, then treated for 1 hour with 1 µM PMA at 37°C. Cells were then fixed and processed for immunofluorescence. Labeled cells were analyzed with an epifluorescence microscope after staining with an anti-L-plastin antibody and Alexa 488-conjugated phalloidin. Scale bar, 10 µm. (C). SiRNA knock-down of PKC-δ decreased PMA-induced L-plastin phosphorylation in MCF-7. MCF-7 cells were transfected with either PKC-δ or PKC-ε siRNAs as well as with negative control siRNA (Ctrl) for 48 hours and then treated with PMA as indicated. Total cell extracts (50 µg) were analyzed by immunoblotting using antibodies specific for Ser5 phosphorylated L-plastin, PKC-ε, PKC-δ and total L-plastin. GAPDH was used to monitor equal protein loading.

We have shown very recently that, in the highly invasive 1001 cells, PKC-δ is the major PKC isozyme responsible for endogenous L-plastin phosphorylation [37]. To identify the PKC isozymes involved in L-plastin phosphorylation in non-invasive epithelial carcinoma MCF-7 cells treated with PMA, we used two different PKC inhibitors GF109203X (specific for α, β1, δ, ε, ζ isozyme inhibition) or Gö6976 (inhibiting α and β1 isozymes) [41]. Interestingly, treatment with GF109203X but not with Gö6976 prevented PMA-induced L-plastin phosphorylation (Fig. 5A).

Accordingly, GF109203X, but not Gö6976, strongly inhibited the formation of ruffling structures and spikes and consequently, the PMA-induced translocation of L-plastin to these structures (Fig. 5B). A role for the atypical PKC-ζ could be excluded at the outset, since unlike classical and novel PKC isozymes, atypical PKC isozymes do not respond to phorbol esters [42]. These results argue in favor of an involvement of novel PKC isozymes (δ and/or ε) in L-plastin phosphorylation and actin cytoskeleton reorganization in response to PMA.

### PKC-δ Is the Major Kinase Involved in PMA-Enhanced L-Plastin Ser5 Phosphorylation

Although the majority of the non-conventional PKC isozymes are expressed in MCF-7 cells [43], it is the novel PKC-δ isozyme that has been extensively described as a regulator of the actin cytoskeleton in epithelial cells [44], [45]. To further dissect the contribution of novel PKC isozymes in PMA-triggered L-plastin phosphorylation, we performed knock-down experiments using siRNA technology resulting in 85% of PKC-δ or 71% of PKC-ε knock-down. The knock-down of PKC-δ significantly inhibited PMA-dependent L-plastin phosphorylation in MCF-7 cells (63% decrease of the amount of phosphorylated L-plastin), whereas PKC-ε knock-down had no significant effect (Fig. 5C). Accordingly, knock-down of PKC-™, but not of PKC-ε considerably impaired the formation of ruffling membranes and spike-like structures (data not shown). These findings suggest that PKC-™ is the major PKC isozyme involved in PMA-triggered signaling pathways leading to L-plastin phosphorylation. Altogether our results point to a role for PKC-δ signaling in the regulation of L-plastin activity in MCF-7 carcinoma cells.

## Discussion

L-plastin is an actin filament bundling protein which contributes to cancer cell invasion in a phosphorylation-dependent manner. In the present study, we have shown that L-plastin associates with the cytoskeleton following a two-binding-state model, in support of biochemical and structural models. Phosphorylation on Ser5 increased its association rate and, hence, appears to promote the L-plastin capacity to dock efficiently to the actin cytoskeleton and regulate actin turn-over. In support of this hypothesis, we have provided evidence for the first time that L-plastin modulates actin dynamics in focal adhesions and increases their F-actin content by decreasing the actin dissociation rate at actin filament ends, this effect being also promoted by L-plastin Ser5 phosphorylation. In carcinoma cells, consistent with the role of L-plastin in controling actin dynamics, the PKC activator PMA induced the translocation of L-plastin to *de novo* actin polymerization sites in ruffling membranes and protruding spike-like structures, and significantly enhanced its phosphorylation on Ser5 via PKC-δ signaling pathways. In agreement with these findings, we identified L-plastin as a component of a protein complex comprising cortactin, a major regulator of actin dynamics. Altogether, our data support a role for L-plastin in the control of actin turn-over which is modulated by its phosphorylation on residue Ser5. In MCF-7 carcinoma cells, invasion-promoting pathways appear to upregulate L-plastin phosphorylation in parallel to actin cytoskeleton reorganization.

In our FRAP assays, the recovery of GFP-L-plastin fluorescence fitted a two-binding-state model [31], comprising a first quick and a second slow binding phase. Such a mechanism would be in good agreement with previous data suggesting that the two ABDs of L-plastin have non-identical interactions with F-actin [20] and that ABD2 binds actin first, enabling the subsequent binding of ABD1 to a second filament [14]. Phosphorylation on residue Ser5 was proposed to regulate the targeting of L-plastin to the actin cytoskeleton [9], [33]. Here we found that the non-phoshorylatable S5/A-L-plastin variant exhibited a lower association rate with focal adhesions in Vero cells than WT-L-plastin, in line with its weak colocalization with actin-rich structures, as reflected by the large pool of unbound S5/A-L-plastin in the cytosol. Phosphorylation of L-plastin mainly increased the association rates k*_1on_ and k*_2on_ and decreased the concentration of free unbound L-plastin molecules *F_eq_*. Although our experimental set-up does not allow to determine the order of binding of the two ABDs, our FRAP data favor a mechanism in which phosphorylation regulates the docking of the protein to the cytoskeleton rather than its dissociation. Interestingly, the two ABDs have been described as being packed tightly together in an approximately antiparallel arrangement. Since a subset of the presumed actin-binding sequences within the ABDs face toward the ABD1-ABD2 interface, a conformational rearrangement appears to be required for their participation to F-actin binding [13]. Whether L-plastin phosphorylation contributes to such a conformational change enabling efficient F-actin binding remains to be investigated.

Importantly, our data support a role for L-plastin in the control of actin dynamics and turn-over which is modulated by L-plastin phosphorylation. Indeed, all L-plastin variants decreased the pool of free actin monomers *F_eq_* and the actin dissociation rate *k_off_* in focal adhesions when compared with those of control cells, indicating the presence of more F-actin. Several mechanisms may account for the decrease in actin filament turn-over. L-plastin may, by binding along actin filaments, protect them against depolymerization as supported by previous *in vitro* data [20]. This effect might not necessary rely on actin bundling since binding of the single ABDs of L-plastin and its closely related T-plastin isoform, were sufficient for decreasing actin depolymerization *in vitro* [20], [46]. Alternatively, L-plastin may protect actin filaments against disassembly by cofilin, as previously proposed for T-plastin [46]. The observed effect on actin turn-over was clearly more pronounced for the phosphorylatable WT- or the phosphomimetic S5/E-L-plastin variants than for the unphosphorylatable S5/A-L-plastin. L-plastin phosphorylation may have a direct effect on actin filament stabilization by enhancing L-plastin binding along their side and/or L-plastin-mediated bundling, a mechanism which is supported by previous biochemical data [9]. Such a mechanism of regulation has also been reported for ABP-280, an actin-binding protein that binds and crosslinks actin filaments more readily when tyrosine phosphorylated [47]. Most interestingly, the S5/E-L-plastin variant induced even more strikingly varying *K_eq_*, k*_on_ and *F_eq_* values for actin than WT-L-plastin as compared to non-transfected control cells. These observations suggest that the negatively charged glutamate residue, mimicking Ser5 phosphorylation and escaping inactivation through dephosphorylation, might keep the S5/E variant locked in a high affinity state. Thus phosphorylation-dephosphorylation cycles of L-plastin might be coupled to actin dynamics, as also supported by our results obtained in MCF-7 cells. A similar, yet opposite phosphorylation-dependent fascin activity has been proposed to regulate filopodial dynamics, since an unphosphorylatable fascin mutant was constitutively active and enhanced filopodia formation and length, whereas a phosphomimetic variant acted as a dominant negative mutant [26]. Compared to the actin-bundling protein fascin, L-plastin may have a more pleiotropic function since it associates with various actin-rich structures, including ruffling membranes, in cells.

As L-plastin Ser5 phosphorylation has been correlated with the progression to an invasive cell phenotype [8], there is growing interest in studying the effect of L-plastin phosphorylation in epithelial cancer cells. PMA, a potent PKC activator, is known to have profound effects on MCF-7 breast carcinoma cell morphology and motility [48], and to contribute to the invasive behaviour of MCF-7 cells [49]. Here, we have shown that PMA treatment of MCF-7 cells leads to L-plastin phosphorylation and cytoskeletal rearrangements with concurrent L-plastin translocation to newly formed spikes and ruffling membranes in these cells. Inhibition assays using PKC isozyme-specific inhibitors have highlighted a role for novel PKC isozymes in PMA-triggered L-plastin phosphorylation and actin cytoskeleton remodeling, although the contribution of other kinases is not necessarily excluded. More precisely, our siRNA knock-down studies have revealed that PKC-δ signaling pathways are necessary for PMA-induced L-plastin phosphorylation. Notably, constitutive L-plastin phosphorylation in the highly invasive MCF-7-derived 1001 cells has also been shown to depend predominantly on the novel PKC-δ isozyme [37]. In hematopoietic cells, the role of PKCs remains highly controversial [22], [24], [50]. Whereas recent work has shown the involvement of a distinct subset of PKC isozymes in L-plastin phosphorylation in neutrophils upon N-formyl-L-methionyl-L-leucyl-L-phenylalanine stimulation [25], most of the past studies made no distinction between the different PKC isozymes. Altogether our results suggest that, in carcinoma cells, PKC-δ signaling is involved in L-plastin phosphorylation, an event that appears to link signal transduction pathways and cytoskeletal dynamics. However, it is important to note that a direct L-plastin phosphorylation by PKC-δ or by any other PKC isoform could not be demonstrated *in vitro* [51].

PMA treatment of MCF-7 cells induced a profound change in cell morphology with concomitant L-plastin translocation to *de novo* assembled actin-rich structures. Based on its actin filament bundling activity, we had expected L-plastin to be recruited to F-actin structures containing tightly bundled actin filaments such as stress fibers. However, L-plastin associated barely with these structures and mainly targeted to sites of active actin polymerization such as the proximal part of protruding spikes or ruffling membranes. While microspikes and filopodia contain aligned actin filaments, ruffling membranes harbour a dendritic network of actin (reviewed in [52]). Thus, unlike other bundling proteins such as fascin [26], L-plastin appears to be able to associate with both types of actin organization, dendritic networks and aligned filaments. Notably, recent results using L-plastin nanobodies have demonstrated that L-plastin bundling activity is necessary to maintain filopodial integrity [53]. An implication of L-plastin in controling actin dynamics is supported by our observation that cortactin, an Arp2/3 complex-binding protein [54]–[56], was found in a complex with phosphorylated L-plastin in PMA-treated MCF-7 cells. It is noteworthy that cortactin could also be associated with protein complexes containing unphosphorylated L-plastin depending on the experiment. Thus, phosphorylation of L-plastin does not appear to be strictly required for protein complex formation, although it might stabilize such complexes. Localization of L-plastin to ruffling membranes and its presence in a complex comprising cortactin links this protein to the active remodeling of actin filaments where it may contribute to stabilize the filaments. In support of this mechanism, the T-plastin isoform has also been reported to stabilize actin filaments and modulate Arp2/3-mediated actin assembly in an *in vitro* reconstitution assay [46].

Taken together, our results quantitatively demonstrate for the first time that L-plastin affects actin dynamics and turn-over in live cells, an effect which is modulated by its phosphorylation on Ser5. Although phosphorylation of L-plastin does not appear to be absolutely required for its recruitment to actin polymerization sites, it might, by increasing the pool of high affinity L-plastin, contribute to the fine-tuning of actin turn-over. Our study paves the way for future investigations of the role of L-plastin in carcinoma cell invasion as a modulator of actin turn-over.

## Materials and Methods

### Ethics

An ethics statement is not required for this work.

### Cell Culture

Monkey kidney Vero cells were grown in DMEM (Dulbecco's modified Eagle's medium). The human breast carcinoma MCF-7 cell line was grown in RPMI (Roswell Park Memorial Institute) medium. Media were supplemented with 10% fetal bovine serum. Cells were grown at 37°C, under 5% CO_2_ atmosphere.

### Antibodies and Reagents

Polyclonal rabbit IgGs against L-plastin and serine-5 phosphorylated L-plastin (anti-Ser5-P) have been characterized before [3], [9]. Mouse monoclonal anti-GFP and anti-cortactin antibodies were obtained from Sigma (Bornem, Belgium) and from Millipore (Billerica, MA), respectively. Rabbit polyclonal anti-PKC-δ and rabbit monoclonal anti-PKC-ε were from Cell Signaling Technology, Inc. (Danvers, MA). Mouse anti-glyceraldehyde-3-phosphate dehydrogenase (GAPDH), Rhodamine-conjugated phalloidin and Alexa 488-conjugated phalloidin were purchased from Molecular Probes (Invitrogen, Merelbeke, Belgium). Texas red- and Cy2 green-conjugated secondary antibodies were from Jackson ImmunoResearch Laboratories (De Pinte, Belgium). The anti-rabbit and anti-mouse IgG antibodies coupled to horseradish peroxidase were purchased from Amersham, GE Healthcare (Diegem, Belgium). Phorbol 12-myristate 13-acetate (PMA) and cytochalasin D (CytoD) were obtained from Sigma, the PKC inhibitors GF109203X and Gö6976 from Calbiochem (Leuven, Belgium) and lipofectamine 2000 from Invitrogen.

### Construction of DNA Constructs

pDsRed-Monomer-N1 vectors (Takara Bio-Europe/Clontech, Saint-Germain-en-Laye, France) containing wild-type- (WT), unphosphorylatable Ser5/Ala (S5/A) or phosphomimetic Ser5/Glu (S5/E)-L-plastin were generated from previously described pGEX-2T-WT-L-plastin, pGEX-2T-S5/A-L-plastin and pGEX-2T-S5/E-L-plastin vectors, respectively [9]. Briefly, WT-, S5/A-, or S5/E-L-plastin 1880-bp *Eco*RI-restricted cDNA fragments were inserted into DsRedN1 *Eco*RI-cut vectors. The correct orientation of inserts was verified by sequencing. pEGFP-C vectors (Clontech) containing WT-, S5/A-, or S5/E-L-plastin were generated from pDsRedN1-WT-L-plastin, pDsRedN1-S5/A-L-plastin, or pDsRedN1-S5/E-L-plastin vectors, respectively. Briefly, the pEGFPC2-S5/A-L-plastin was cloned by *Eco*RI/*Bam*HI restriction of the pDsRedN1-S5/A-L-plastin vector. The resulting fragment was inserted into the *Eco*RI/*Bam*HI-restricted pEGFPC2 vector. The pEGFPC2-WT- or -S5/E-L-plastin vectors were constructed by substitution of the 5′-end of the pEGFPC2-S5/A-L-plastin vector by the 5′-end of WT- or S5/E-L-plastin, obtained by *Eco*RI/*Sca*I restriction of pDsRedN1-WT- or -S5/E-L-plastin vectors, respectively. All constructs were verified by sequencing.

### Recombinant Proteins

GST and GST-fusion proteins were produced in *E. coli* from the pGEX-2T expression vector and purified as described previously [17]. The concentration of thrombin-cleaved proteins was determined using the Bradford assay (Bio-Rad, Nazareth, Belgium) and by SDS-polyacrylamide gel electrophoresis (PAGE) using a BSA protein standard curve.

### Transient Transfection of Cells

5–10 µg of cDNA encoding L-plastin phosphorylation variants or β-actin were transfected into 5×10^6^ Vero cells by electroporation at 240 V and 950 µF [57]. MCF-7 cells were transfected using lipofectamine 2000. For knock-down experiments, cells were seeded at a density of 5×10^4^ cells per well in a 6-well plate 24 hours prior to transfection. siRNA transfection was performed using lipofectamine 2000 according to the manufacturer's protocol. Double stranded 21-mers validated siRNA for PKC-δ, -ε and negative control were purchased from Qiagen (Venlo, The Netherlands). The mRNA and protein levels of siRNA targeted genes were analyzed 48 hours after transfection by RT-qPCR and immunoblotting, respectively. Quantification of siRNA-mediated knock-down of PKC-δ and PKC-ε was performed by densitometric scanning of the autoradiograms (hp scanjet 5470c and ImageJ software). The percentage of PKC-δ or PKC-ε expression was calculated as the ratio between PKC-δ or PKC-ε expression after PKC-δ or PKC-ε siRNA transfection respectively versus their expression after control siRNA transfection (100%).

### Treatment of Cells with Pharmacological Agents

MCF-7 cells were pretreated for 3 hours in the presence or absence of PKC inhibitors and incubated for 1 hour with or without 1 µM PMA. In some experiments, MCF-7 cells were pretreated for 1 hour with 0.5 µM cytochalasin D and then incubated 1 hour with or without 1 µM PMA. Cells were then processed either for indirect immunofluorescence or for cell lysis.

### Indirect Immunofluorescence

Cells were washed with PBS supplemented with 0.1 mM CaCl_2_ and 0.1 mM MgCl_2_, fixed with 3% paraformaldehyde and processed for immunofluorescence labeling as described previously [58]. Labeled cells were analyzed by epifluorescence microscopy (Leica DMRX microscope) or a Zeiss laser scanning confocal microscope (LSM-510 Meta, Carl Zeiss, Jena, Germany). Images were acquired with a linear CCD camera (Micromax, Princeton Instruments, Trenton, NJ) and analyzed with Metaview software (Universal Imaging Corporation Ltd., Buckinghamshire, UK).

### Immunoblotting

Cells were lysed for 30 minutes in ice-cold RIPA buffer (10 mM Tris-HCl pH 7.4, 150 mM NaCl, 0.1% SDS, 1% Triton X-100, and 1% Na-deoxycholate) containing a cocktail of protease inhibitors (Roche Diagnostics GmbH, Mannheim, Germany). Lysates were cleared by centrifugation at 20000×*g* for 10 minutes at 4°C. The total protein concentration was determined using the Bradford assay. Total cell lysates (50 µg of proteins) were separated by SDS-PAGE and transferred onto nitrocellulose membrane (Amersham, GE Healthcare) using a semi-dry transblot apparatus. Primary antibodies were revealed using secondary antibodies coupled to horseradish peroxidase and enhanced chemiluminescence (ECL) detection method. Quantification of L-plastin phosphorylation was performed by densitometric scanning of the autoradiograms obtained using the anti-Ser5-P antibody (hp scanjet 5470c and ImageJ software). The percentage of L-plastin phosphorylation was expressed as the ratio of phosphorylated L-plastin after PKC isozyme-specific siRNA transfection versus phosphorylated L-plastin after control siRNA transfection (100%).

### Pull-Down and Immunoprecipitation

GST or GST-fusion proteins (20 µg), immobilized on glutathione-sepharose™ 4B beads (Amersham, GE Healthcare), were incubated overnight with cell extracts (200 µg) on an end-over-end rotor at 4°C. The bead pellet was rinsed three times with cold PBS, then resuspended in 2 x SDS-containing sample buffer and boiled for 5 minutes at 95°C.

For immunoprecipitation, 10^6^ cells transiently transfected with expression vectors encoding GFP or WT GFP-L-plastin were homogenized in 500 µl lysis buffer (10 mM Tris-HCl, pH 7.5, 100 mM NaCl, 0.5 mM EDTA, 1 mM PMSF, 0.5% Nonidet P-40). After a centrifugation step of 10 minutes at 20000×*g* at 4°C, the supernatant was adjusted to 1 ml with dilution buffer (10 mM Tris-HCl, pH 7.5, 150 mM NaCl, 0.5 mM EDTA, 2 mM PMSF). 20 µl (2%) were added to SDS-containing sample buffer and used for SDS-PAGE (referred to as input). 30 µl of GFP-nanotrap beads [40] were added and incubated for 30 minutes on an end-over-end rotor at 4°C. After a centrifugation step of 2 minutes at 2700×*g* at 4°C, the supernatant was removed, and 20 µl (2%) of the supernatant were used for SDS-PAGE (referred to as flow-through). The bead pellet was washed three times with 500 µl dilution buffer. After the last washing step, the beads were resuspended in 2 x SDS-containing sample buffer and boiled for 10 minutes at 95°C.

The obtained samples were analyzed by SDS-PAGE with subsequent Coomassie Blue staining or immunoblotting.

### FRAP Experiments

FRAP experiments were performed with a Zeiss LSM510 Meta laser scanning confocal microscope. Transfected Vero cells were kept at 37°C using an air-stream incubator-XL and a heating frame (Carl Zeiss). The excitation wavelength and emission filters were 488 nm/band-pass 505 to 530 nm and 543 nm/long-pass 560 nm for GFP and DsRed respectively. Image processing was performed using Zeiss LSM510 Image Browser version 4.0.

For FRAP experiments, a 63x/1.4 NA oil-immersion objective was used, and the confocal pinhole was set to 2.5 µm. Prebleach and recovery images were acquired at a rate of 1 image/second. We intentionally used the rather long time step (1 s) in order to acquire fluorescence recovery over the optimal time range (90 s) for investigation of L-plastin binding and actin polymerization properties. For photobleaching, all argon laser lines (458, 477, 488, and 514 nm) were used simultaneously at 100% transmittance for 3 iterations to bleach a circular area of 5 µm diameter, the region of interest (ROI). Normalized FRAP curves were generated from raw data as described [59]. Briefly, intensity in the bleached region (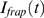) and in the whole cell (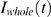) at each time point are initially subtracted by the corresponding background intensity (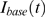). These intensities are then rescaled to the average prebleach intensities in the corresponding regions (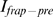 and 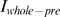). The normalized FRAP curve (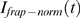) is the ratio of FRAP and whole cell rescaled intensities. The resulting equation is
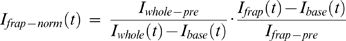
(1)


To fit the normalized recoveries, we applied the models of binding reactions in a reaction dominant regime; a one-binding-state or a two-binding-state [31]. The general chemical rate equation for a single binding reaction is
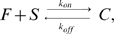
(2)where *F* represents a free protein, *S* is a vacant binding site, *C* denotes bound [FS] complexes, and *k_on_* and *k_off_* are the association and dissociation rates respectively. Assuming that the biological system is in equilibrium and the number of binding sites is constant, then the fluorescence intensity within the bleached spot is
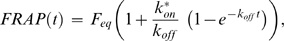
(3)where *F_eq_* is the equilibrium normalized concentration of free monomers, the pseudo-association rate k*_on_ is the product of the association rate, *k_on_* and the steady-state concentration of vacant binding sites, *S_eq_* (k*_on_ = k_on_·*S_eq_*) [59]. From the values of model parameters, the ratio of bound to free molecule concentrations (*K_eq_*) is calculated as 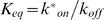.

In a two-binding-state model, the chemical rate equations are
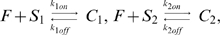
(4)where subscripts *1* and *2* refer to the different binding states. Using similar assumptions as for the one-binding-state model the total fluorescence intensity is
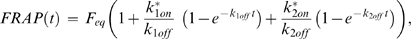
(5)where k*_1on_, k*_2on_, k_1off_, *k_2off_* are the pseudo-association and -dissociation rate constants at the first and second binding states, respectively [31].

FRAP curves were fitted with equations (3) or (5), yielding the parameters *F_eq_*, k*_on_ and *k_off_*, k*_1on_ and *k_1off_*, k*_2on_ and *k_2off_*. All fitting procedures were performed with *Nonlinear Regress* function in Mathematica 6 (Wolfram Research).
